# Inhibition of Allograft Inflammatory Factor-1 in Dendritic Cells Restrains CD4^+^ T Cell Effector Responses and Induces CD25^+^Foxp3^+^ T Regulatory Subsets

**DOI:** 10.3389/fimmu.2017.01502

**Published:** 2017-11-08

**Authors:** Diana M. Elizondo, Temesgen E. Andargie, Dazhi Yang, Apollo D. Kacsinta, Michael W. Lipscomb

**Affiliations:** ^1^Department of Biology, Howard University, Washington, DC, United States; ^2^Department of Cellular and Molecular Medicine, UCSD School of Medicine, La Jolla, CA, United States

**Keywords:** dendritic cells, T regulatory cells, tolerogenic, polarization, suppression

## Abstract

Allograft inflammatory factor-1 (AIF1) is a cytoplasmic scaffold protein shown to influence immune responses in macrophages and microglial cells. The protein contains Ca^2+^ binding EF-hand and PDZ interaction domains important for mediating intracellular signaling complexes. This study now reports that AIF1 is expressed in CD11c^+^ dendritic cells (DC) and silencing of expression restrains induction of antigen-specific CD4^+^ T cell effector responses. AIF1 knockdown in murine DC resulted in impaired T cell proliferation and skewed polarization away from T helper type 1 and 17 fates. In turn, there was a parallel expansion of IL-10-producing and CD25^+^Foxp3^+^ T regulatory subsets. These studies are the first to demonstrate that AIF1 expression in DC serves as a potent governor of cognate T cell responses and presents a novel target for engineering tolerogenic DC-based immunotherapies.

## Introduction

Dendritic cells (DC) are professional antigen presenting cells that direct T cell activation, proliferation, and polarization (1, 2). In addition to directing immunity, DC also play prominent roles in modulating peripheral tolerance by inducing anergic states in responder T cells and/or directing fates toward T regulatory cell (Treg) states (3–5). The biological factors and mechanisms that govern direction toward immunity vs. tolerance by DC are not fully understood.

Allograft inflammatory factor-1 (AIF1), also known as ionized calcium-binding adapter molecule 1, is a 17 kD interferon gamma-inducible calcium-binding EF-hand protein (6, 7). The gene, situated in the major histocompatibility class III genomic region (8), has demonstrated diverse roles in both the nervous and immune systems (9–13). For immune cells, it is largely restricted to the monocyte and macrophage lineages. In particular, expression of AIF1 in macrophages has been shown to promote pro-inflammatory responses (7, 14–16). Similarly, expression in microglial cells (17), which are derived from the macrophage lineage (18), is important in modulation of synaptic activities and upregulated in response to nerve damage (19). Dysregulation of AIF1 expression has been largely associated with both neuroinflammatory- and autoimmune-related disorders (13, 20, 21). Although reports have shown the importance of AIF1 in macrophage and microglial lineages, no study has determined its role in DC.

In this study, AIF1 function in DC antigen presentation was studied using RNA interference approaches. Results reveal that AIF1 expression in DC supports direction of T cells toward immunity. Silencing of AIF1 in DC induced CD4^+^ T cells toward Tregs and away from IL-17- and IFNγ-producing T helper cells. This is the first study to clearly define the presence of AIF1 in DC and its functional role in governing antigen-specific T helper cell responses. Taken together, this investigation provides evidence that AIF1 expression in DC is instrumental in promoting adaptive immune response and that its loss leads to tolerance.

## Materials and Methods

### Mice

Mice were purchased from Jackson Laboratory and bred in-house. All animal procedures performed were approved by the Institutional Animal Care and Use Committee. Femurs and tibias were harvested from C57BL/6 [wild-type (WT)] mice between 8 and 14 weeks of age to generate bone marrow-derived DC. WT mice were also used as recipients for *in vivo* adoptive transfer experiments. Transgenic B6.Cg-Tg(TcraTcrb)425Cbn/J (OT-II) mice were used as a source of naive CD4^+^ T cells responsive to ovalbumin (OVA_323–339_).

### Generation of Bone Marrow-Derived DC and Small Interfering RNA (siRNA) Knockdown

Bone marrow-derived DC were generated as described by a modified protocol of Inaba et al. (22). Briefly, bone marrow cells were cultured in IMDM (Thermo Fisher Scientific, Grand Island, NY, USA) supplemented with 10% fetal bovine serum (FBS; Thermo Fisher Scientific), 2 mM L-glutamine (Thermo Fisher Scientific), 100 U/ml penicillin/streptomycin (Thermo Fisher Scientific), and 20 ng/ml GM-CSF for 8 days in culture. On day 6 (of the 8-day culture), cells were purified for a homogenous DC population using CD11c microbeads (Miltenyi Biotec, Auburn, CA, USA) for positive selection. AIF1 was knocked down using an ECM 830 (BTX, Holliston, MA, USA) square wave electroporator with 1 nmol (6.65 µg) of siRNA oligos in 4 mm gap cuvettes with the following settings: 310 V, 10 ms, 1 pulse. AIF1 siRNA (siAIF1) sequence used: 5′-GGCAAGAGAUCUGCCAUCUUG-3′ (Thermo Fisher Scientific, Grand Island, NY, USA). Scrambled siRNA served as controls (siControl): 5′-GGGCTCTACGCAGGCATTTAA-3′. Additionally, studies used silencer pre-designed siRNA 73668 targeting AIF1 purchased from Thermo Fisher Scientific: 3′-GGUGAAGUACAUGGAGUUU-5′. After electroporation of siRNA on day 6 in CD11c^+^-sorted DC, cells were placed back into culture. On day 7, 24 h after siRNA transfection, DC were matured with 250 ng/ml of LPS (or other TLR agonists) for an additional 24 h. On day 8, these siRNA transfected mature DC were used to assess immunophenotype and prime naïve CD4^+^ OT-II T cells. For all *in vivo* studies, DC were adoptively transferred into mice 24 h after siRNA transfection to compensate for the trafficking time required to enter the draining lymph nodes and prime T cell responses.

### Isolation of CD4^+^ T Cells for *In Vitro* Stimulation and CFSE Proliferation Assays

For isolation of naïve CD4^+^ T cells from OT-II mice, CD8^+^ cytotoxic T cells and MHC class II^+^ antigen presenting cells were depleted by negative selection from spleen and lymph nodes using primary antibodies to CD8 and MHC class II (BioLegend, San Diego, CA, USA) followed by secondary labeling with anti-rat IgG magnetic microbeads (Qiagen, Hilden, Germany). Cells were then depleted by passing through a magnetic column. The approach yielded 96 ± 2.1% purity of CD4^+^ T cells. These naïve CD4^+^ T cells were cultured with 1.0, 0.3, or 0.1 µg/ml of OVA peptide (ISQAVHAAHAEINEAGR)-_323–339_-pulsed siAIF1 or siControl LPS-matured DC at a ratio of 10:1, respectively. Peptides were purchased from AnaSpec (Fremont, CA, USA). Scrambled non-specific peptides served as controls for some experiments, with the following sequences: VAAGIAQAHESIREHAN and IENHQIAGAAERSAAVH.

OVA_323–339_-pulsed siAIF1 or siControl mature DC stimulated OT-II CD4^+^ T cells were harvested at the 24 h time point to evaluate early activation markers CD69, CD62L, and CD25; antibodies purchased from BioLegend. For proliferation assays, CD4^+^ T cells pre-labeled with 2.5 µM CFSE (Thermo Fisher Scientific) were cultured with OVA_323–339_-pulsed siAIF1 or siControl DC for 96 h. Cells were co-stained with antibodies to IL-2 (BioLegend) for intracellular cytokine detection after fixation and permeabilization. For polarization experiments, OVA_323–339_-pulsed siAIF1 or siControl DC were cultured with CD4^+^ T cells for 12–14 days with re-stimulation on day 5 using respective peptide-pulsed siAIF1 or siControl DC supplemented with 200 U/ml of IL-2. T cell cytokine responses were then evaluated by stimulation with 20 ng/ml PMA and 1 µg/ml ionomycin for 4 h in the presence of 10 µg/ml of brefeldin A prior to fixation, permeabilization, and intracellular staining of IFNγ, IL-4, IL-17A, and IL-10. For Treg phenotype, cells were stained 12–14 days after initial priming by OVA_323–339_-pulsed siAIF1 or siControl DC for CD25, Foxp3, CD27, CTLA-4, and CD44. These cells were not stimulated with mitogens prior to immunophenotyping. All antibodies purchased from BioLegend. Cells were then acquired by a flow cytometric analyzer.

### Treg *In Vitro* Suppression Assays

OT-II T cells were expanded for 12–14 days by siAIF1 or siControl DC pulsed with OVA peptide. After expansion, these T cells were then labeled with Cell Tracker Violet dye (Thermo Fisher Scientific). These labeled cells are referred to as *suppressors*. Next, naïve CD4^+^ or CD8^+^ T cells were isolated from WT mice by negative depletion and confirmed CD25^−^ by flow cytometric analyses. Briefly, the CD4^+^CD25^−^ or CD8^+^CD25^−^ T cells were labeled with 2.5 µM CFSE. These CFSE-labeled naïve T cells are the *responders*. The purpose of labeling with different dyes was to distinguish *suppressor* from the *responder* populations. The *suppressor* and *responder* T cells were then cultured together at a 3:1, 1:1, 1:3, and 1:10 ratio, respectively, prior to stimulation with anti-CD3/CD28-coated microbeads (Dynabeads; Thermo Fisher Scientific). Cells were incubated for 72–96 h prior to collection, staining, and analysis of *responder* T cell proliferation using a modified approach by Collison and Vignali (23).

### *In Vivo* Adoptive Transfer of DC and Assessment of T Cell Responses

CD4^+^ T cells were isolated from OT-II mice and labeled with CFSE. Next, 5 × 10^6^ of these CFSE-labeled OT-II CD4^+^ T cells were intravenous injected into WT mice, as described by Moon et al. (24). Next, 2 × 10^6^ control (siControl) or AIF1 knockdown (siAIF1) DC pulsed with OVA_323–339_ were subcutaneously (s.q.) injected once at 6 h or twice at 6 and 12 h after the transfer of the CFSE-labeled OT-II cells. For these studies, DC were treated with siRNA 24 h prior to adoptive transfer, with LPS stimulation at the 12 h time point. Finally, the DC were incubated with 5 µg/ml of OVA_323–339_ peptide in the last 4 h prior to adoptive transfer. Subcutaneous injection sites were the scruff of the neck and/or the upper thigh-just above the hind leg. The draining lymph nodes nearest to the s.q. injection were harvested 3.5 or 5.5 days post-injection to assess CD4^+^ T cell proliferation and effector responses. In each experiment, the same lymph nodes were collected: axillary, brachial, inguinal, lumbar, and superficial cervical. The tissues were then disassociated into single suspension, washed, fixed, and stained for intracellular assessment of cytokines.

### Cytometric Bead Array Multiplex Analysis and ELISA

After various time points of DC or DC-T cell co-culture incubation periods, cells were spun down and supernatants collected. Mouse Inflammation Kit Cytometric Bead Array (CBA; BD Biosciences) and Legendplex Mouse Th Cytokine Panel (BioLegend) were used to detect cytokine levels in the supernatant; CBA kit analyzed IL-12p70, IL-10, IL-6, TNFα, MCP-1, and Legendplex was used to analyze IL-17A, IL-4, and IFNγ. Median fluorescence intensity values were normalized to cytokine concentration based on internal standard controls following manufacturer recommended protocols.

### Cryosection, Staining, and Microscopy

Lymph nodes and spleens from WT mice were harvested and immediately fixed in 3% paraformaldehyde in PBS overnight at 4°C. After fixation, tissues were placed in 10% sucrose for 1 h prior to cryosectioning at 25 and 50 µm. Next, sections were permeabilized and stained with CD11c-Alexa488 (BioLegend), rabbit monoclonal AIF1 (EPR16588 or EPR178847; Abcam, Cambridge, MA, USA), and DAPI. Secondary anti-rabbit Alexa-594 was used to detect AIF1. Sections were then imaged using a three-channel wide-field fluorescence microscope and analyzed by ImageJ.

### Western Blotting

Cell lysates were prepared by incubating Nonidet P-40 cell lysis buffer (Amresco, Solon, OH, USA) with cells for 30 min before high-speed centrifugation. Lysates were collected and ran in 15% gels using vertical gel electrophoresis. Protein content was transferred from gels to nitrocellulose blots using the Pierce Power Blotter (Thermo Fisher Scientific). GAPDH served as a loading control. After primary antibody staining, secondary antibodies conjugated to fluorochromes were used for visualizing protein bands on the Odyssey imaging system (LI-COR, Lincoln, NE, USA). Image Studio 5.2 software (LI-COR) was used to calculate relative fluorescence intensities.

### Flow Cytometry

Cell surface staining was performed in PBS supplemented with EDTA and 2.5% FBS (FACS buffer). Single cell suspensions were washed with FACS buffer two to three times prior to staining with fluorochrome tagged-antibodies. Cells were stained for 15 min at 4°C with 10 µl of a 10 µg/ml working concentration per 2 × 10^5^ cells. Cells were then washed and fixed for 20 min in 3% paraformaldehyde at 4°C. For intracellular staining, fixed cells were permeabilized with 0.2% saponin in PBS for 1 h. Next, primary antibodies were added and cells incubated for 1 h. For primary unconjugated antibodies, secondary-tagged fluorochrome-labeled antibodies were prepared. These secondary antibodies were diluted to 1:1,000–1:3,000 working concentrations and 10 µl were added per 2 × 10^5^ cells. Cells were allowed to incubate for 1 h or overnight followed by extensive washing. Samples were acquired using a BD FACSVerse flow cytometric analyzer. Datasets were analyzed using FlowJo v10 (TreeStar, Ashland, OR, USA). Respective isotype controls and/or fluorochrome-labeled isotype controls were used in all assays. Gating strategies were established based on respective isotype controls.

### Statistical Analysis

GraphPad Prism v6.0 (GraphPad Software, La Jolla, CA, USA) was used to determine statistical significance. Student unpaired two-tailed *t*-test was used to evaluate the significance of two groups. One-way or two-way analysis of variance was used to evaluate the significance between the means of three or more independent groups. A *p*-value ≤0.05 was considered statistically significant; *<0.05, **<0.01, and ns, non-significant. Error bars for all figures indicate SEs.

## Results

### AIF1 Is Expressed in DC

**Figure 1:**
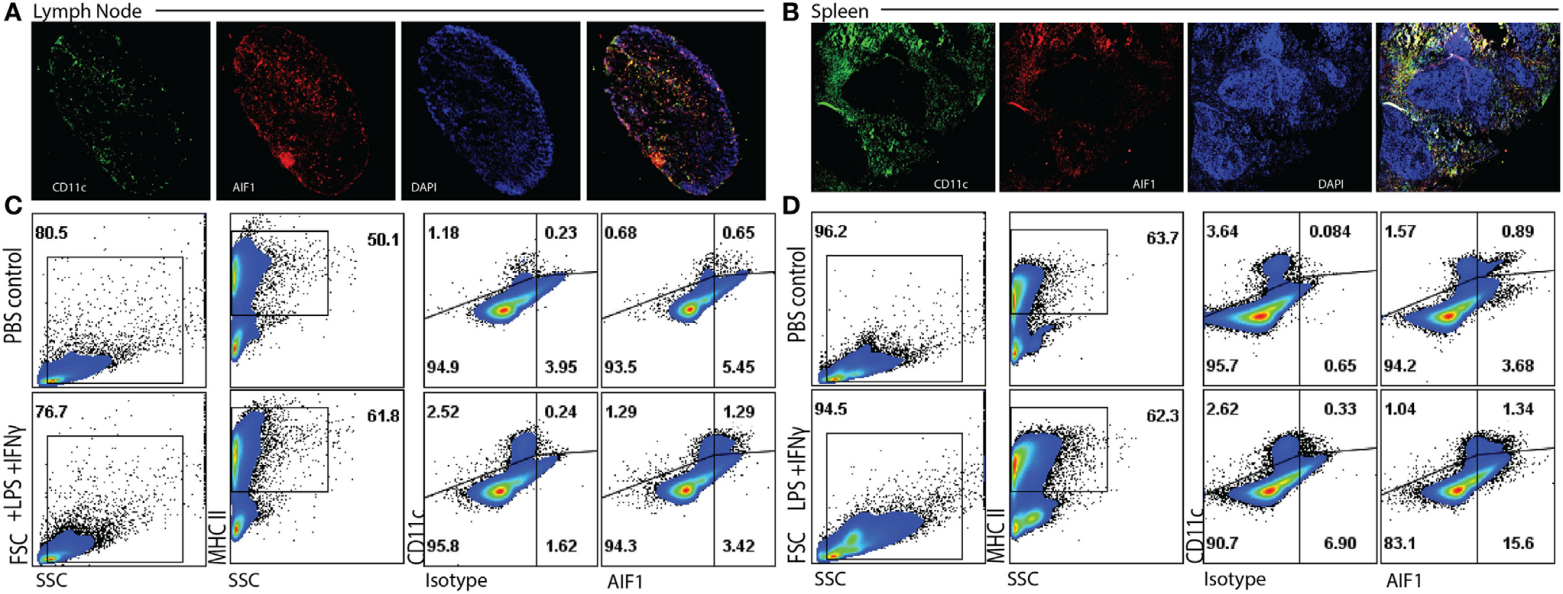
Allograft inflammatory factor-1 (AIF1) is expressed in splenic and lymph node dendritic cells. **(A)** Lymph nodes and **(B)** spleen were harvested from wild-type (WT) mice prior to cryosectioning, fixation, and staining for CD11c (green), AIF1 (red), and nucleus (DAPI; blue). Cells were imaged at 5× for lymph nodes and 7× for spleen using a wide-field fluorescent microscope. WT mice were treated with PBS (as control) or LPS and IFNγ for 24 h. **(C)** Lymph nodes and **(D)** spleens were harvested and disassociating into single cell suspension prior to fixation and staining for AIF1, MHC class II, and CD11c. Flow cytometric analysis was performed by gating on live MHC class II^+^ subsets and assessing CD11c vs. AIF1 expression; isotype controls were used to establish gating strategies for AIF1 expression. Data are representative of three independent experiments.

To assess expression of AIF1 in DC, both lymph nodes and spleens were harvested from WT mice and evaluated for co-expression with CD11c^+^ subsets. WT mice were injected with either PBS or an LPS and IFNγ cocktail for 24 h. Spleen and lymph node tissues were then isolated, cryosectioned, and stained. Fluorescence microscopy revealed co-localization of AIF1 with CD11c^+^ cells in both lymph nodes and the spleen (Figures 1A,B). Next, total cells isolated from the lymph nodes or the spleen were analyzed by flow cytometry for expression of AIF1. Live cells were gated on MHC class II and evaluated for co-expression of CD11c and AIF1. Results revealed high expression of AIF1 in MHC class II^+^CD11c^+^ DC from both lymph nodes and the spleen (Figures 1C,D). Finally, LPS and IFNγ stimulation resulted in significant increase in AIF1 expression in these MHC class II^+^CD11c^+^ subsets.

**Figure 2:**
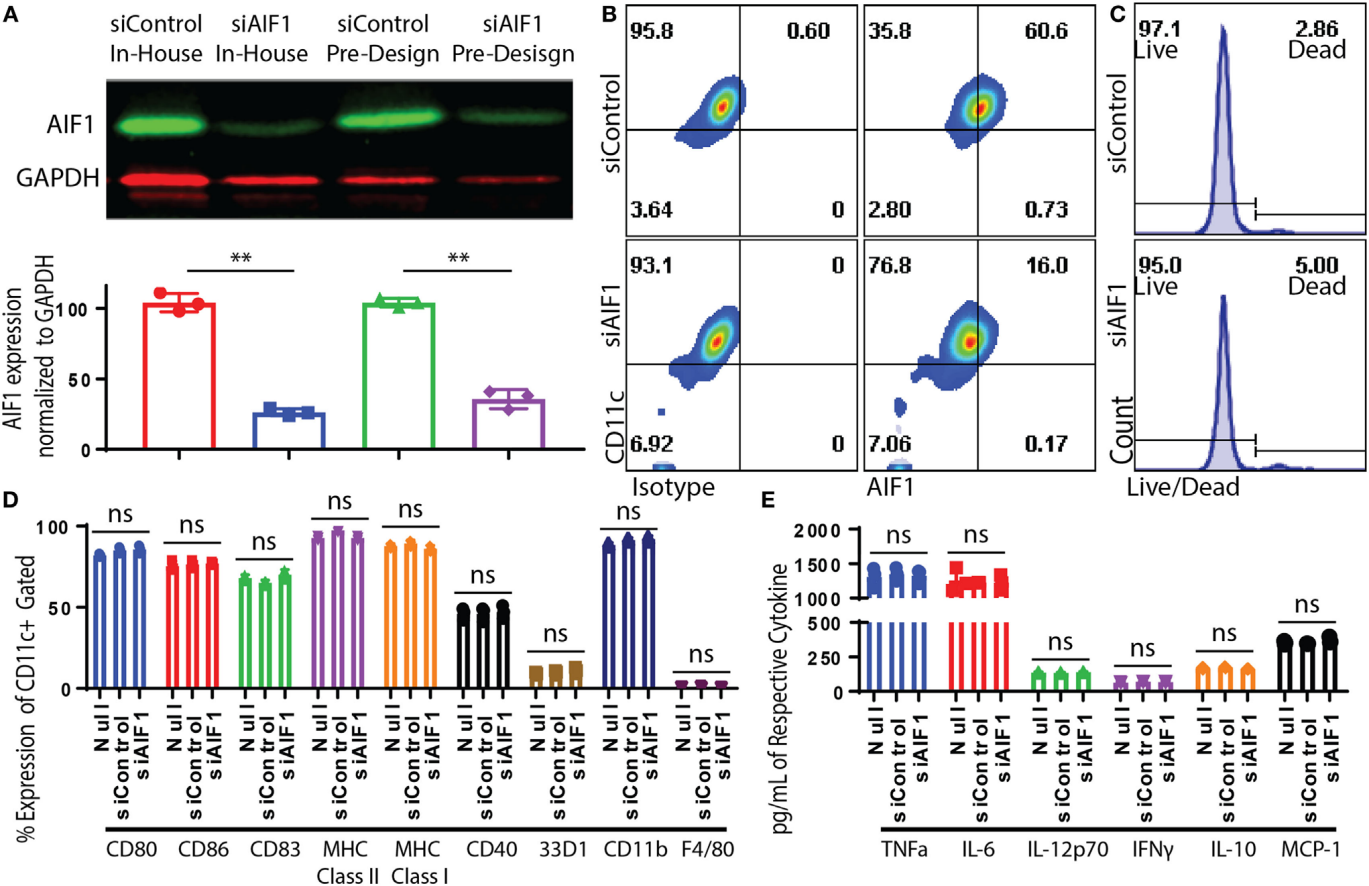
Dendritic cells (DC) expression of allograft inflammatory factor-1 (AIF1) knocked down by small interfering RNA (siRNA). Bone marrow-derived DC were sorted for CD11c^+^ subsets by magnetic bead-positive selection. CD11c^+^ DC were knocked down with two different siRNA oligos targeting AIF1 (siAIF1). In-house designed siRNA oligos were designed in the laboratory to target exon 6 of AIF1. Pre-design siRNA oligos were commercially available from Thermo Fisher Scientific and target exon 5. Scrambled sequence of both siRNAs was generated to serve as controls (siControl). **(A)** Knockdown efficiency was assessed by western blot and bar graph displays AIF1 expression normalized to GAPDH loading control. siControl in-house designed is red (●), siAIF1 in-house designed is blue (■), siControl pre-design is green (▲), and siAIF1 pre-design is purple (♦). Data are representative of three replicate groups. **(B)** Knockdown of AIF1 in DC was further evaluated by flow cytometric analysis; isotype controls were used to establish all gating parameters. Datasets represent three replicate groups from three independent experiments. **(C)** Viability of DC after siRNA knockdown was assessed using a live/dead cell fluorescence staining kit. Data are representative of three independent experiments. **(D)** CD11c^+^ siAIF1 or siControl DC were matured with 250 ng/ml of LPS for 24 h prior to immunophenotyping. Untransfected mature DC (null) were included as controls. Cells were stained for CD80, CD86, CD83, MHC class II, MHC class I, CD40, 33D1, CD11b, and F4/80. **(E)** Supernatant from LPS-matured null, siAIF1, or siControl DC groups were assessed for TNFα, IL-6, IL-12p70, IFNγ, IL-10, and MCP-1 using cytometric bead array assays. Median fluorescence intensity was correlated with cytokine concentrations using standard internal controls following the manufacture recommended protocol. A p-value ≤0.05 was considered statistically significant; *<0.05, **<0.01, and ns, non-significant.

Bone marrow-derived DC were generated and sorted to yield a pure population of CD11c^+^ subsets (Figure S1A in Supplementary Material). Transfection of siRNA oligonucleotides targeting AIF1 (siAIF1) was used to knockdown the gene expression in CD11c^+^ DC. Scrambled non-targeting siControl. Results yielded a consistent 70–75% knockdown of AIF1 from endogenous levels as measured by western blot analyses (Figure 2A). Flow cytometric analyses confirmed that siRNA targeted knockdown of AIF1 in the CD11c^+^ DC reduced expression from 60.6 down to 16.0% 48 h post-transfection (Figure 2B). Results also revealed that optimal siRNA knockdown of AIF1 occurred during the 48–72 h time point post-transfection and that sustained depression of AIF1 lasted up to 5 days in culture (Figure S1B in Supplementary Material). Knockdown using siRNA (both in-house and pre-design oligonucleotides) did not yield any major differences in viability in comparison with controls (Figure 2C). Phenotypic characterization of LPS-matured CD11c^+^ DC transfected with siAIF1 showed no significant differences in expression of CD80, CD86, CD83, CD40, 33D1, CD11b, MHC class I, MHC class II, and F4/80 compared with siControl treated (Figure 2D). Furthermore, no significant differences were found in secretion of the pro-inflammatory cytokines TNFα, IL-6, IL-12p70, IFNγ, IL-10, and MCP-1 (Figure 2E). These observations were recapitulated using other TLR agonists (i.e., TLR3-Poly I:C, TLR5-Flagellin, TLR9-CpG; data not shown).

### Loss of AIF1 in DC Impairs Antigen-Specific T Cell Responses

**Figure 3:**
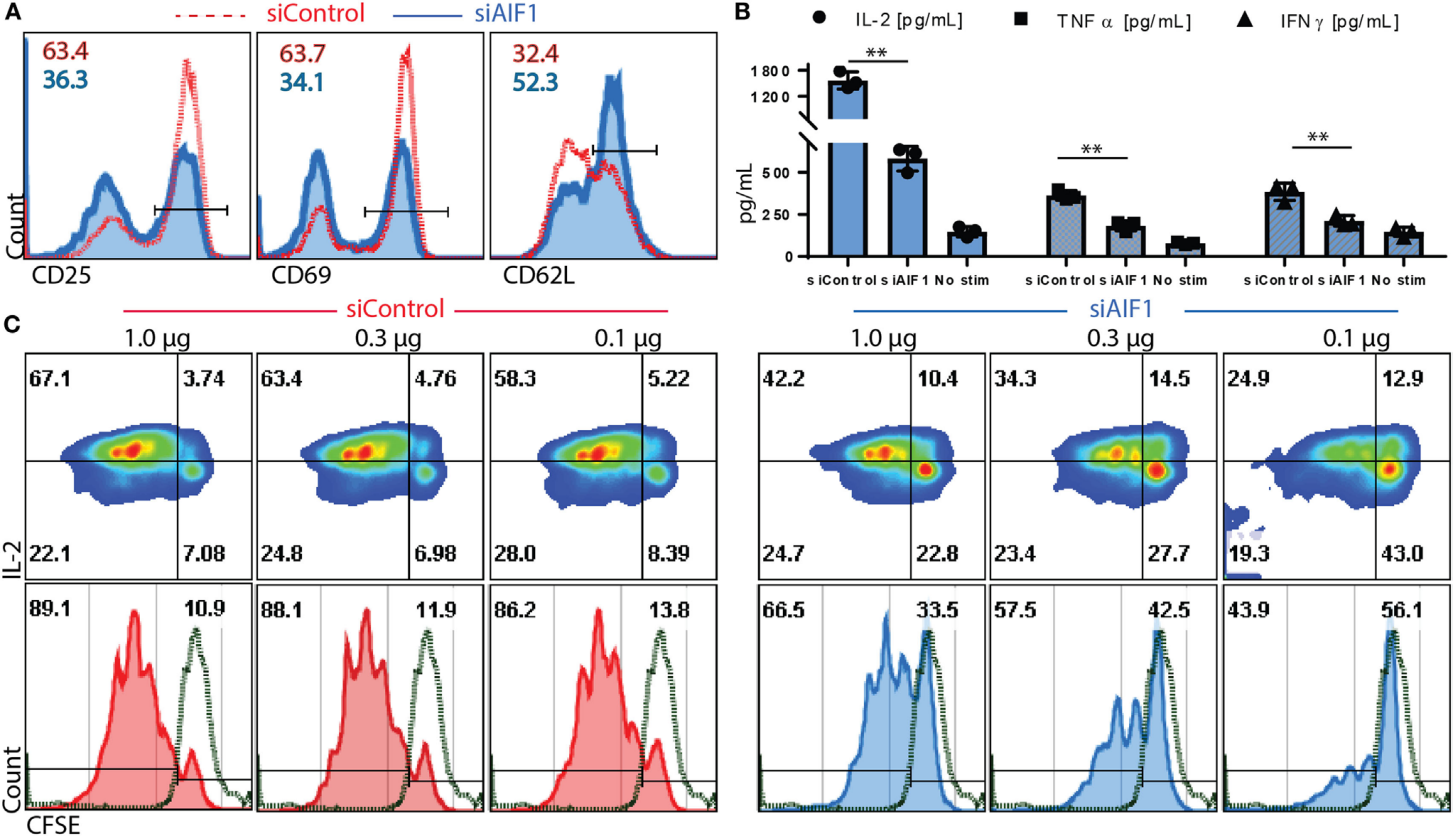
Allograft inflammatory factor-1 (AIF1) knockdown dendritic cells (DC) have impaired ability to prime early activation and proliferation of responder CD4^+^ T cells. AIF1 knockdown (siAIF1) or control treated (siControl) DC were LPS-matured and pulsed with OVA_323–339_ peptide prior to culturing with naïve CD4^+^ T cells from OT-II mice. **(A)** After 24 h of culture of naïve CD4^+^ T cells with siControl of siAIF1 DC, cells were harvested and stained for CD25, CD69, and CD62L to measure early activation. siControl primed T cells (red dashed line) are overlayed on the siAIF1 cohort (blue solid line). **(B)** Supernatant was harvested from the co-culture at the 72 h time point and measured for IL-2 (●), TNFα (■), and IFNγ (▲) using cytometric bead array assays. Median fluorescence intensity was correlated with cytokine concentrations using standard internal controls following manufacture recommended protocol. The no stim group represents T cells cultured in the absence of either siAIF1 or siControl DC. **(C)** CD4^+^ OT-II T cells were labeled with CFSE prior to culturing with siControl or siAIF1 DC pulsed with 1.0, 0.3, or 0.1 µg/ml of OVA_323–339_ peptide for 96 h. Cells were harvested and co-stained with IL-2. Left panel represents siControl (red) and the right panel siAIF1 (blue) OVA-pulsed DC cultured with responder CFSE-labeled CD4^+^ T cells. Dot plot shows IL-2 on the *y*-axis vs. CFSE on the *x*-axis. Histogram plot below each dot plot shows CFSE vs. count to best delineate proliferation stages. Green dashed line represents non-stimulated CFSE-labeled T cells (in the absence of siAIF1 or siControl OVA-pulsed DC). Gates were established using non-stimulated T cells. Data are representative of four independent experiments in three replicate wells. A *p*-value ≤0.05 was considered statistically significant; *<0.05, **<0.01, and ns, non-significant.

Antigen presentation capacity of DC was evaluated upon AIF1 knockdown. In these studies, siControl vs. siAIF1 LPS-matured DC pulsed with OVA_323–339_ peptide was cultured with naïve OT-II CD4^+^ T cells. These isolated T cells were confirmed to be naïve CD4^+^ CD25^−^ subsets (Figure S1C in Supplementary Material). After 24 h, impaired early activation responses were observed in cognate T cells primed by siAIF1 DC compared with siControl, as measured by reduced CD25 (63.4–36.3%) and CD69 (63.7–34.1%) expression and restrained down regulation of CD62L (32.4–52.3%) (Figure 3A). Supernatant harvested at 72 h of culture was also assessed. These studies found decreased levels of IL-2, TNFα, and IFNγ in the CD4^+^ T cells primed by AIF1 knockdown DC compared with siControl (Figure 3B); the no stimulation group contained T cells without the presence of OVA peptide-pulsed DC. Lastly, CFSE-labeled CD4^+^ T cell responders were primed by siControl or siAIF1 DC at varying concentrations of peptide (1.0, 0.3, and 0.1 μg/ml) and allowed to incubate for 96 h prior to measurement. Cells were then harvested and co-stained with antibodies targeting IL-2. The results revealed a significant reduction in proliferative capacity of the siAIF1 DC primed T cells compared with the siControl group (Figure 3C); non-stimulated CFSE-labeled T cells served as internal controls. There was a significant impairment in T cell proliferative capacity in the siAIF1 cohort, reducing proliferation by as much as 50% at 0.1 µg/ml of OVA peptide vs. siControl DC groups. T cell responses were found to be antigen dose dependent, where higher levels of peptide stimuli could override the phenotype observed in the siAIF1 DC stimulated groups. To confirm antigen-specificity, scrambled OVA peptide was employed as an additional internal control. No proliferation occurred in the scrambled OVA control from either siAIF1 or siControl DC stimulated groups (Figure S1D in Supplementary Material).

**Figure 4:**
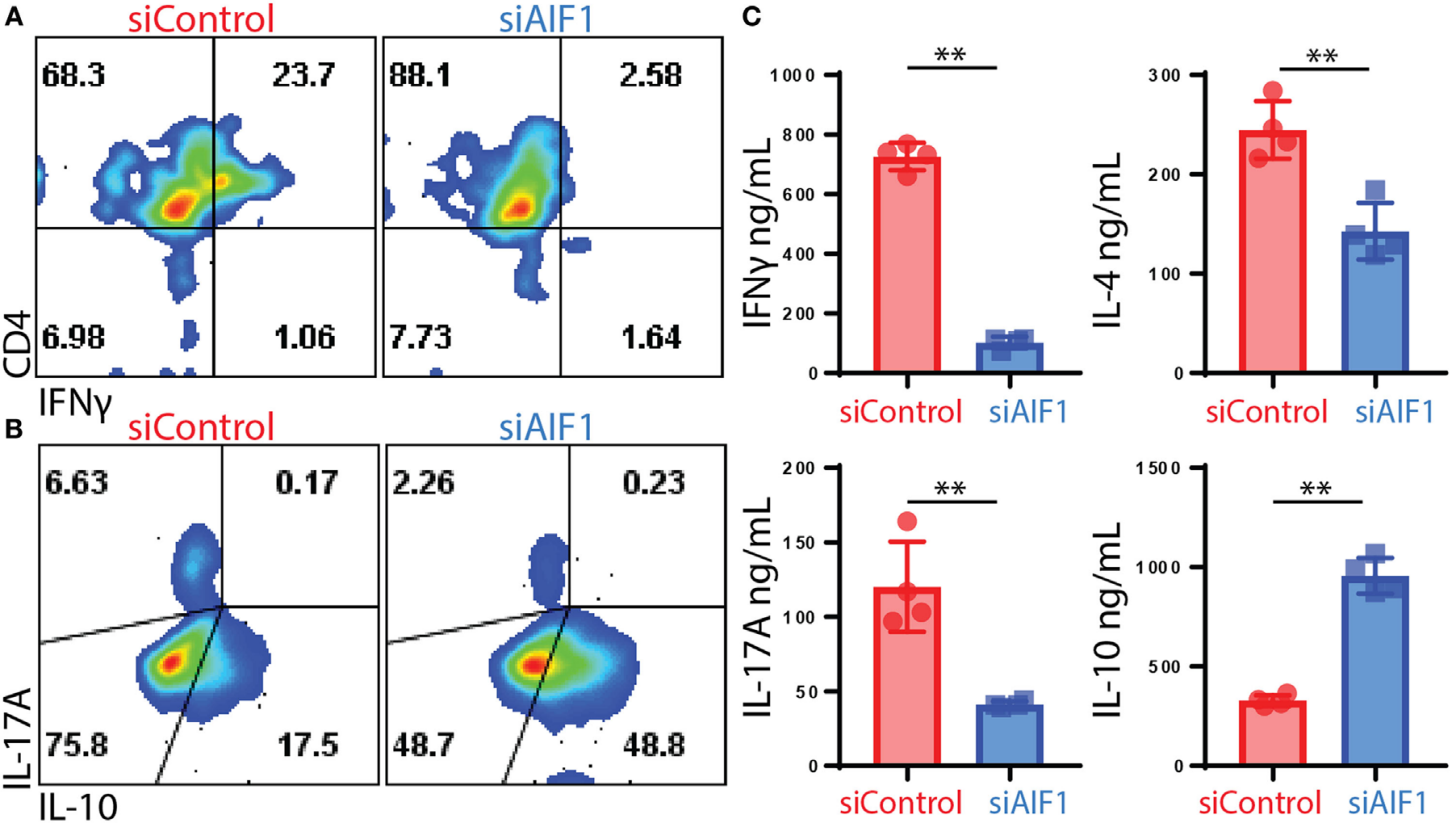
Restrained T cell cytokine production of IL-17A and IFNγ upon stimulation by allograft inflammatory factor-1 (AIF1) knockdown dendritic cells (DC). AIF1 knockdown or control LPS-matured CD11c^+^ DC were used to prime naïve CD4^+^ T cells from OT-II mice for 12 days in culture. T cells were then stimulated with PMA/ionomycin in the presence of brefeldin A for 4 h prior to intracellular staining of CD4, IFNγ, IL-17A, and IL-10. **(A,B)** Results show dot plots of CD4 vs. IFNγ and IL-17A vs. IL-10 in siControl vs. siAIF1 DC primed OT-II CD4^+^ T cells. **(C)** Additionally, 4 h after PMA/ionomycin stimulation, supernatant was collected and assessed for IFNγ, IL-4, IL-17A, and IL-10 by cytometric bead array. Bar graph presents siControl as red (●) and siAIF1 as blue (■). Data are representative of four independent experiments in three replicate wells. A *p*-value ≤0.05 was considered statistically significant; *<0.05, **<0.01, and ns, non-significant.

For evaluating polarization responses, T helper cell-associated cytokine expression was measured 12 days after initial stimulation by OVA-pulsed siAIF1 vs. siControl DC. These investigations revealed restrained IFNγ from 23.7 to 2.6% expression in siControl vs. siAIF1, respectively, and IL-17A from 6.6 to 2.2% (upon PMA/ionomycin re-stimulation in the presence of brefeldin A; Figures 4A,B). Additionally, in DC primed CD4^+^ T cells, there was a concomitant threefold increase in IL-10 production from 17.5 to 48.8% in siControl vs. siAIF1 DC primed CD4^+^ T cells, respectively (Figure 4B). The datasets are representative of three independent experiments. Intracellular cytokine flow analysis results were further validated using multiplex cytometric bead array assays. Cytokines were measured from supernatant collected 4 h after the PMA/ionomycin re-stimulation. IFNγ, IL-4, and IL-17A cytokine expressions were decreased in siAIF1 group compared with siControl, whereas IL-10 expression was markedly increased (Figure 4C). These findings suggest that AIF1 expression in DC plays an immunomodulatory role in promoting pro-inflammatory responses in T helper cells.

### CD25^+^Foxp3^+^ Tregs Expanded by AIF1 Knockdown DC Are Functionally Suppressive

**Figure 5:**
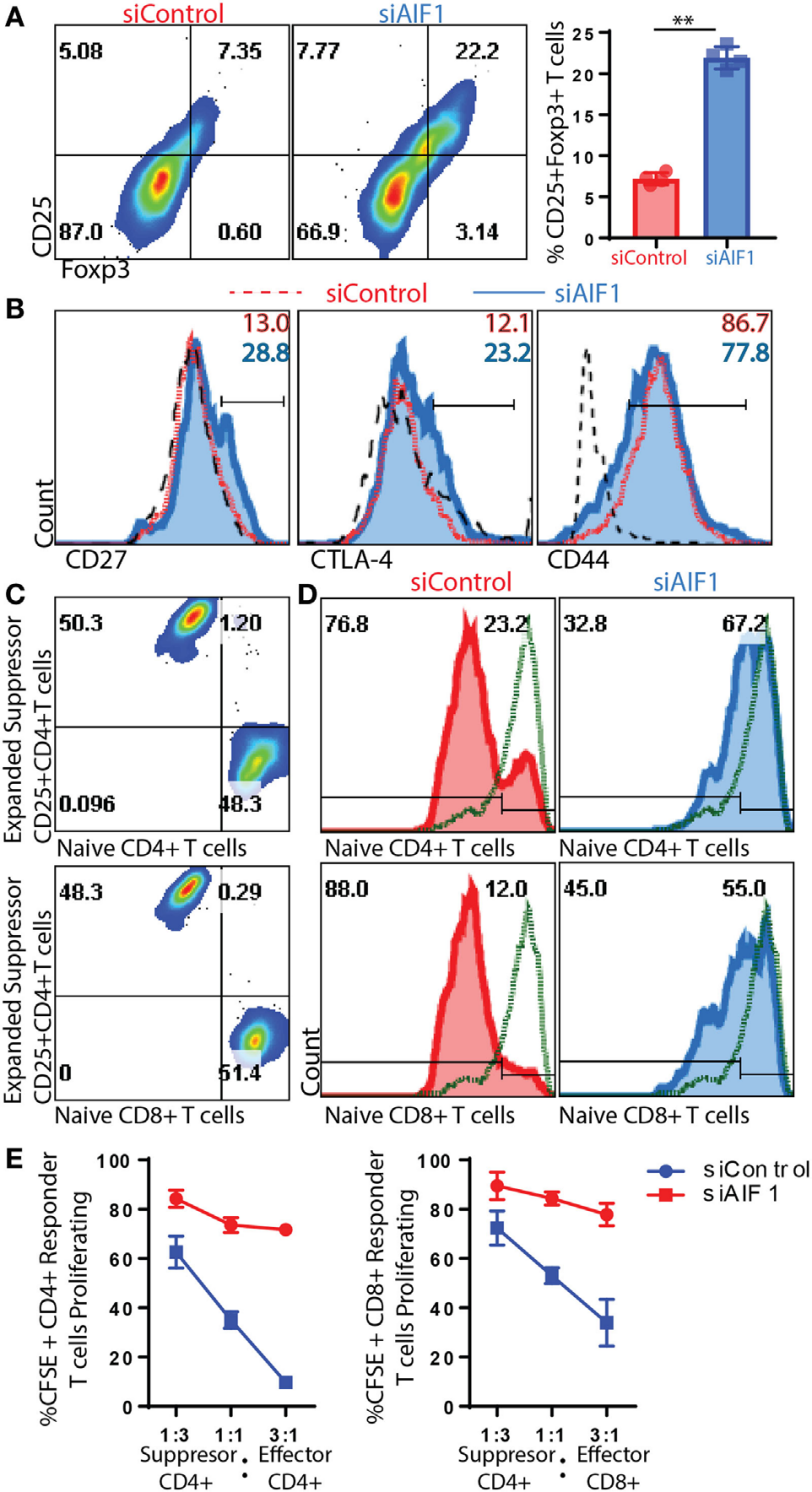
T regulatory cells (Tregs) expanded from allograft inflammatory factor-1 (AIF1) knockdown dendritic cells (DC) are functionally suppressive. AIF1 knockdown or control DC primed naïve CD4^+^ T cells were assessed for generation of CD25^+^Foxp3^+^ Treg cell subsets. **(A)** Twelve to fourteen days after initial priming by siAIF1 or siControl LPS-matured CD11c^+^ DC, T cells were stained with CD4, CD25, and Foxp3. Dot plot shows CD4^+^ gated subsets for CD25 vs. Foxp3. Bar graph to the right of dot plot shows percentage of CD4^+^CD25^+^Foxp3^+^ cells. Red (●) is siControl and Blue (■) siAIF1. **(B)** Analysis of CD27, CTLA-4, and CD44 is shown from siAIF1 vs. siControl DC primed CD4^+^ T cells. Histogram plots shown are from CD4^+^ gated subsets. siControl is red short dashed line unfilled (with percentage positive at the top of each respective histogram) and siAIF1 is blue solid line filled histogram (with percentage positive below). Long black dashed lines are isotype control. **(C)** For Treg suppression assays, the day 14 expanded T cells from siControl or siAIF1 DC stimulated groups were cultured with either CD4^+^ or CD8^+^ T cells freshly isolated from spleen and lymph nodes of wild-type mice. Dot plots show a representation of the siControl DC expanded day 14 CellTracker Violet-labeled suppressor T cells at the top of the *y*-axis. The CFSE-labeled naïve CD4^+^ T cells (top dot plot) or CD8^+^ T cells (bottom dot plot) are positive along the *x*-axis. The differential labeling allows distinguishing between the two populations. **(D)** Histogram plots show proliferation responses of the CFSE-labeled CD4^+^ or CD8^+^ naïve CD4^+^ T cells stimulated with anti-CD3/CD28 in the presence of the day 14- siControl (red; left) or siAIF1 (blue, right) DC expanded CD4^+^ T cells. Top represents proliferation of responder naïve CD4^+^ T cells and bottom the responder naïve CD8^+^ T cells measured. CellTracker Violet-labeled cells were gated out of the presented histogram plots, ensuring that only CFSE-labeled cells were measured for proliferation. Gates were established using non-stimulated CFSE-labeled T cells (green short dashed line unfilled). **(E)** Graph representation of flow cytometric data for percent CFSE positive of siControl DC or siAIF1 DC primed CD4^+^CD25^+^Foxp3^+^ T cells (*suppressor*) cultured at a 1:3, 1:1, or 3:1 ratio with naïve CFSE-labeled CD4^+^ or CD8^+^ T cells (*responders*). All data are representative of at least three independent experiments in three replicate wells. A *p*-value ≤0.05 was considered statistically significant; *<0.05, **<0.01, and ns, non-significant.

Due to increased IL-10 and decreased levels of IFNγ and IL-17A, the studies next measured Treg generation. siAIF1 DC expanded OT-II CD4^+^ T cells resulted in a threefold increase in CD25^+^Foxp3^+^ subsets (7.35–22.2%) compared with siControl DC by day 14 (Figure 5A). Further evaluation found a consistent, albeit moderate, increase in CD27 and CTLA-4, as well as a decrease in CD44 in the siAIF1 group compared with siControl (Figure 5B). This corroborated literature reports that Foxp3^+^ cells are largely present within the CD27^+^ population and that CTLA-4 is associated with attenuation of antigen presenting cells *via* B7 co-stimulatory engagement (25–27).

Subsequent experiments assessed whether these T cells expanded by siAIF1 DC were functionally suppressive. Briefly, total OT-II CD4^+^ T cells expanded from siControl or siAIF1 DC were harvested, washed, and labeled with a violet marker; this group is termed the *suppressors*. These *suppressors* were then counted and cultured with newly isolated naïve WT CD4^+^ or CD8^+^ T cells labeled with CFSE at approximately a 3:1, 1:1, and 1:3 ratio; these CFSE-labeled naïve WT T cells are termed the *responders* (Figure 5C). This approach allowed distinguishing between the two populations to distinctly assess proliferation of each respective group. Upon anti-CD3/CD28 stimulation, both CFSE-labeled CD4^+^ and CD8^+^ T cell *responders* cultured with siAIF1 expanded *suppressors* were restrained in proliferation after 72 h in culture (Figures 5D,E). In three independent experiments, results identified markedly reduced proliferation of *responder* T cells in the presence of siAIF1 expanded *suppressor* T cells compared with that of the siControl group. This confirmed that the siAIF1 expanded CD4^+^ Tregs were functionally suppressive in their ability to restrain neighbor T cell expansion.

### Adoptive Transfer of AIF1 Knockdown DC Restrains T Effector Responses *In Vivo*

**Figure 6:**
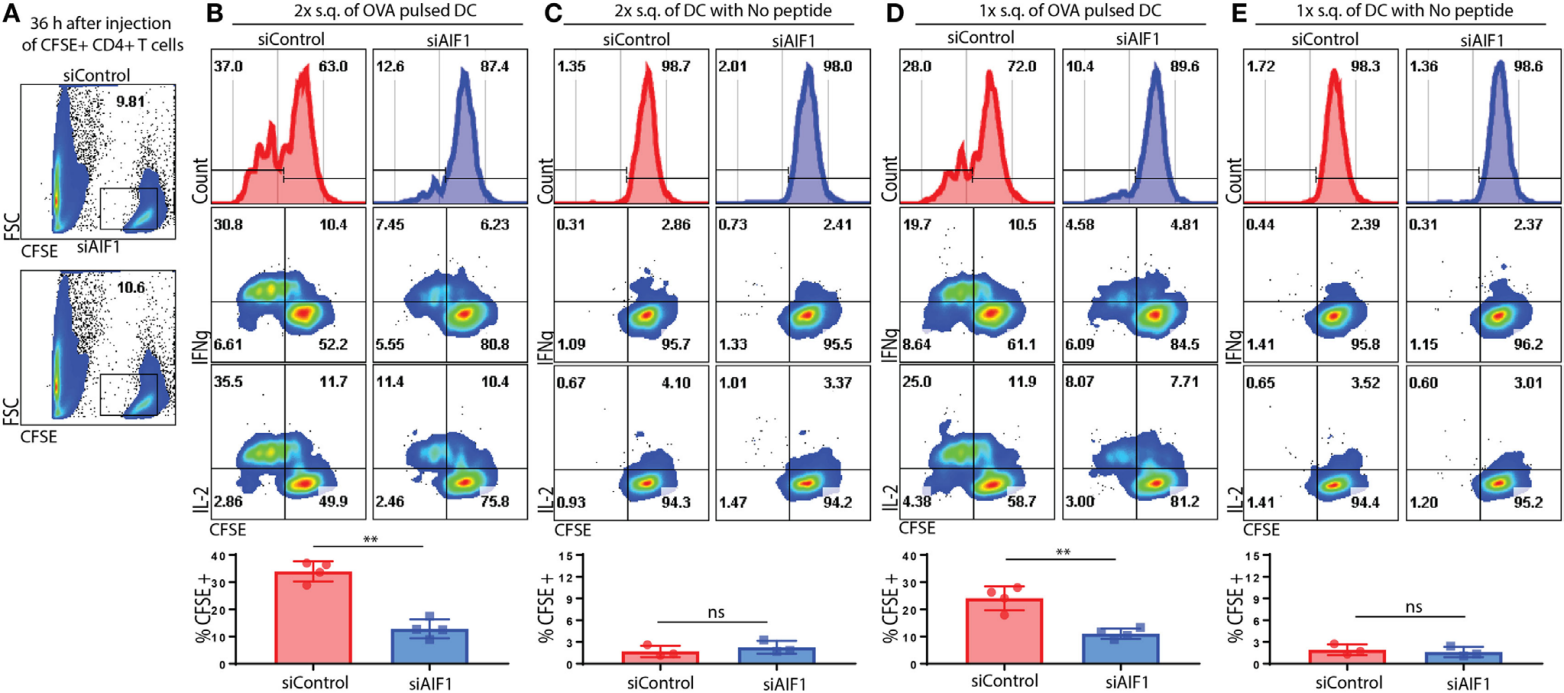
Adoptive transfer of allograft inflammatory factor-1 (AIF1) knockdown dendritic cells (DC) restrains T cell proliferation and IFNγ production *in vivo*. OT-II CD4^+^ T cells were harvested from spleen and lymph nodes prior to labeling with CFSE. These OT-II CFSE-labeled CD4^+^ T cells were then intravenously injected into wild-type (WT) recipient mice. Next, OVA_323–339_-pulsed LPS-matured control (siControl) or AIF1 knockdown (siAIF1) DC were then subcutaneously injected once (1×) at 6 h or twice (2×) at 6 and 12 h after initial injection of the OT-II CFSE-labeled CD4^+^ T cells into the respective WT recipient mice. **(A)** Thirty-six hours after intravenous injection of OT-II labeled CD4^+^ T cells into WT recipients, tissues were harvested to assess frequency of CFSE^+^ cells. *x*-axis is FSC and *y*-axis is CFSE. **(B,D)** After 5.5 days post OT-II CFSE-labeled CD4^+^ T cell followed by the siAIF1 or siControl DC adoptive transfers, mice were sacrificed and draining lymph nodes harvested. Cells were washed, fixed, permeabilized, and co-stained with antibodies to IL-2 and IFNγ prior to flow cytometric analysis. Histogram plots (top) show CFSE expression on *x*-axis as a measure of proliferation in the siControl vs. siAIF1 DC adoptive transfer groups. All gates defining the CFSE^+^ T cells were established using isotype and unstained controls. Dot plots show IFNγ (middle) and IL-2 (bottom) co-expression with CFSE. Bar graphs below show percent of proliferating subsets and statistical relevance. **(C,E)** No peptide-pulsed LPS-matured siControl or siAIF1 DC adoptively transferred with OT-II CFSE-labeled CD4^+^ T cells into the WT recipient mice served as internal controls. Data are representative of three independent experiments with three to four mice per group. A *p*-value ≤0.05 was considered statistically significant; *<0.05, **<0.01, and ns, non-significant.

*In vivo* adoptive cell transfer studies were performed to corroborate *in vitro* results. Briefly, CFSE-labeled OT-II CD4^+^ T cells were adoptively transferred into WT recipient mice. Approximately 10% of CFSE^+^ OT-II cells were identified in the lymph nodes 36 h after adoptive transfer into the WT recipients (Figure 6A). siAIF1 or siControl OVA_323–339_-pulsed LPS-matured DC were then injected subcutaneously either once (1×) at 6 h or twice (2×) at the 6 and 12 h time points after initial transfer of the CFSE-labeled OT-II CD4^+^ T cells into the WT recipients. After 5.5 days, the draining lymph nodes of recipient mice were then analyzed for proliferation and co-expression of IL-2 and IFNγ from the adoptively transferred CFSE-labeled OT-II T cells. For the mice cohort receiving adoptively transferred DC twice (at 6 and 12 h time points), results showed restrained T cell proliferation, as assessed by CFSE dilution assays, from 37.0% in the siControl to 12.6% in the siAIF1 transfected groups (Figure 6B). Adoptive transfer of LPS-matured siAIF1 or siControl DC into the WT recipients without OVA peptide pulsing served as control (Figure 6C). For the mice cohort receiving adoptively transferred DC once (at 6 h) after initial transfer of the CFSE-labeled OT-II cells, proliferation decreased from 28.0% for siControl to 10.4% for the siAIF1 cohort (Figure 6D). Adoptively transferred siAIF1 or siControl DC without pulsing with OVA peptide elicited no T cell expansion (Figure 6E). These results describe a novel role of AIF1 in governing DC antigen presentation capacity for directing T cell proliferation and effector responses.

## Discussion

These studies demonstrate that AIF1 is well expressed in dendritic cell and involved in directing cognate T cell responses in an antigen-specific manner. AIF1 is expressed in the lymph nodes and the spleen, but not all cells expressing AIF1 co-localized with CD11c. This was not surprising, as others have shown AIF1 expression in macrophages, microglial cells, and monocytes (16, 28, 29). AIF1 expression in DC was further confirmed by flow cytometric analysis, whereby the approach assessed expression in MHC class II^+^CD11c^+^ subsets. This, coupled with LPS and IFNγ stimulation, helped to identify that both steady state and inflammatory DC express AIF1. IFNγ increased expression of AIF1 in DC corroborated literature reports of similar induction in macrophages (15, 28). Finally, *in vitro* bone marrow-derived DC also expressed AIF1. Although an increase in AIF1 was observed upon DC maturation, this was directly proportional to other associated maturation markers (i.e., CD80, CD86, and CD40).

RNAi-mediated silencing of AIF1 in DC resulted in greater than 70% knockdown. Potential artifacts introduced into our system caused by offsite target effects were limited by using two different siRNA oligos. There were no significant changes in associated DC co-stimulatory markers or pro-inflammatory cytokine production between AIF1 knockdown and control groups. However, these studies found restrained antigen-specific CD4^+^ T cell activation and proliferation upon stimulation by OVA peptide presenting siAIF1 DC. As concentrations of antigen peptide approached lower ends (toward physiological relevant levels), the impaired function of AIF1 knockdown DC in directing T cell responses was more apparent. As AIF1 was able to be suppressed for up to 4–5 days upon transfection of siRNA, the time frame for measuring these T cell responses was optimal, as early activation, and proliferation readout indices fall within this time frame (30).

Although AIF1 expression in DC governed antigen-specific T cell responses, the results did not show specific polarization/skewing of T_H_1 vs. T_H_2 vs. T_H_17, as the cytokines IFNγ, IL-4, and IL-17 were all restrained. However, there was a shift from a pro- to an anti-inflammatory state, whereby siAIF1 DC promoted increased IL-10 expression in responder T cells and expanded functionally suppressive CD25^+^Foxp3^+^ Tregs. During these investigations, evaluating CD25 and Foxp3 12–14 days after primary stimulation with siAIF1 or control DC ensured that expression was not a transient byproduct of initial T cell activation (31). Finally, the suppressive function of siAIF1 DC expanded CD25^+^Foxp3^+^ T cells were evaluated for ability to restrain neighboring T cells proliferative responses. Even with robust stimulation using anti-CD3/CD28 microbeads, the expanded T cells from the siAIF1 DC cohort retrained proliferation of naïve CD4^+^ or CD8^+^ T cells. These results corroborate literature reports in that AIF1 has a dominant role in pro-inflammatory-associated diseases (7, 32, 33).

For the *in vivo* experiments, subcutaneous injection of control and AIF1 knockdown DC each resulted in accumulation in the draining lymph nodes at comparatively equal numbers. This would suggest that AIF1 knockdown does not directly impair the migratory abilities upon adoptive transfer. However, within the lymph node compartment, adoptive transfer of antigen bearing AIF1 knockdown DC into WT recipient mice led to impaired cognate T cell proliferation, as well as restrained IL-2 and IFNγ co-expression, when compared with control DC. This corroborates *in vitro* data of impaired effector T cell responses. Taken together, these studies show that AIF1 expression in DC plays an important immunomodulatory role in governing antigen-specific T cell responses.

## Ethics Statement

This study was carried out in accordance with the recommendations of Animal Welfare Assurance guidelines upheld by the Office of Regulatory Research and Compliance. The protocol for use of animal studies was approved by the Institutional Animal Care and Use Committee (IACUC).

## Author Contributions

DE, TA, DY, AK, and ML contributed in experimental design, performing experiments, data analysis, and writing of the manuscript.

## Conflict of Interest Statement

The authors declare that the research was conducted in the absence of any commercial or financial relationships that could be construed as a potential conflict of interest.

## Abbreviations

AIF1, allograft inflammatory factor-1; DC, dendritic cell; mDC, mature dendritic cell; siRNA, small interfering RNA; siAIF1, AIF1 siRNA knockdown group; siControl, scrambled control siRNA knockdown group; Treg, T regulatory cell; WT, wild type.
