# Supplementary figures and images for: Inhibition of Allograft Inflammatory Factor-1 in Dendritic Cells Restrains CD4+ T Cell Effector Responses and Induces CD25+Foxp3+ T Regulatory Subsets
Source: Front Immunol. 2017 Nov 8;8:1502. doi: 10.3389/fimmu.2017.01502 (PMC5682305; doi:10.3389/fimmu.2017.01502)

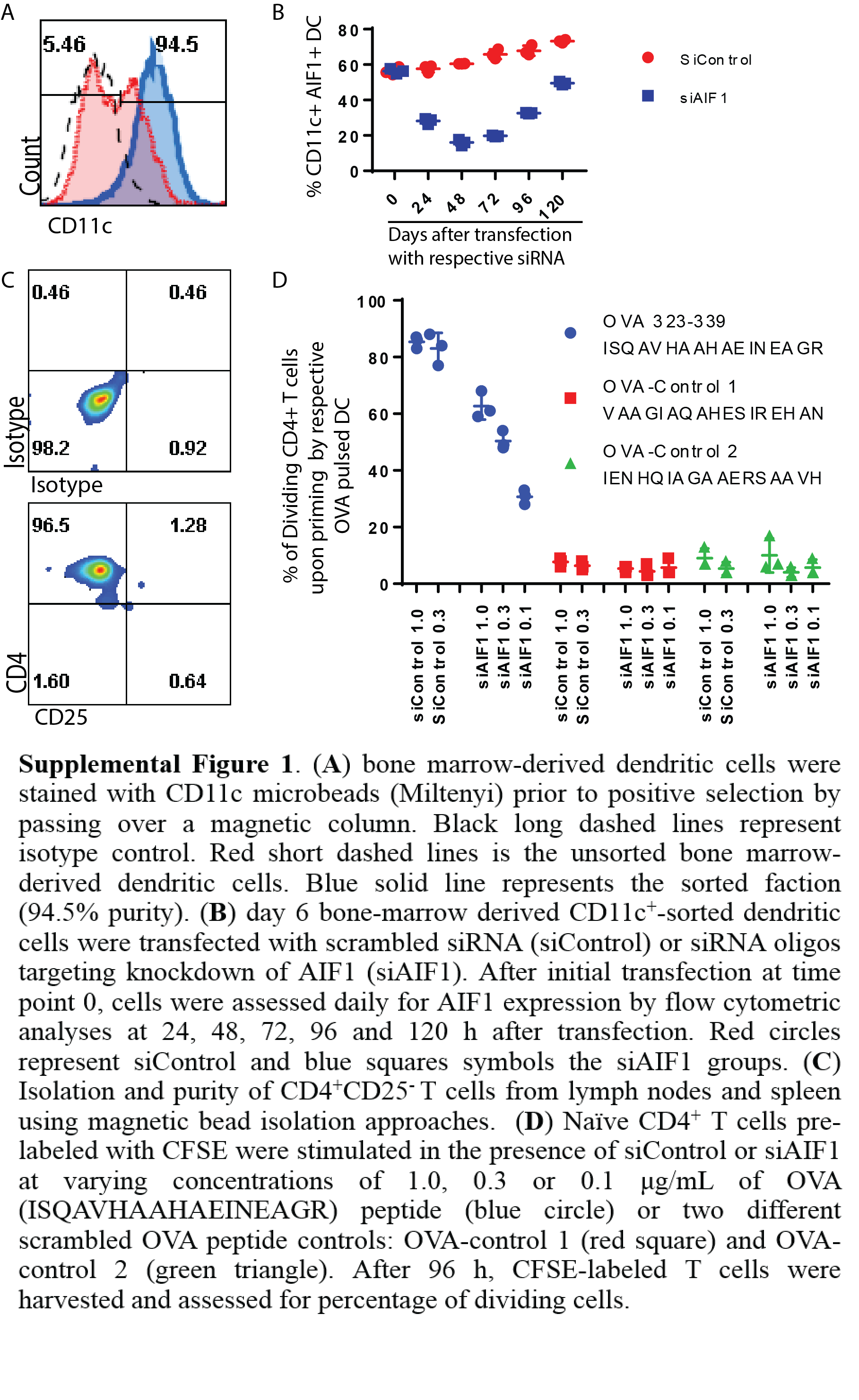

Supplement: Supplementary file 1 [file Image_1.TIF]
